# LBJMR medium: a new polyvalent culture medium for isolating and selecting vancomycin and colistin-resistant bacteria

**DOI:** 10.1186/s12866-017-1128-x

**Published:** 2017-11-23

**Authors:** Lucie Bardet, Stéphanie Le Page, Thongpan Leangapichart, Jean-Marc Rolain

**Affiliations:** URMITE, Aix Marseille Université UM63, CNRS 7278, IRD 198, INSERM 1095 IHU - Méditerranée Infection, 19-21 Boulevard Jean Moulin, 13385 Marseille Cedex 05, France

**Keywords:** Colistin resistance, Culture media, *Mcr-1*, *mcr* genes, Vancomycin-resistant enterococci, *Enterobacteriaceae*, Multi-drug resistant, Detection method, Screening method

## Abstract

**Background:**

Multi-drug resistant bacteria are a phenomenon which is on the increase around the world, particularly with the emergence of colistin-resistant *Enterobacteriaceae* and vancomycin-resistant enterococci strains. The recent discovery of a plasmid-mediated colistin resistance with the description of the transferable *mcr-1* gene raised concerns about the need for an efficient detection method for these pathogens, to isolate infected patients as early as possible. The LBJMR medium was developed to screen for all polymyxin-resistant Gram-negative bacteria, including *mcr-1* positive isolates, and vancomycin-resistant Gram-positive bacteria.

**Results:**

The LBJMR medium was developed by adding colistin sulfate salt at a low concentration (4 μg/mL) and vancomycin (50 μg/mL), with glucose (7.5 g/L) as a fermentative substrate, to a Purple Agar Base (31 g/L). A total of 143 bacterial strains were used to evaluate this universal culture medium, and the sensitivity and specificity of detection were 100% for the growth of resistant strains. 68 stool samples were cultured on LBJMR, and both colistin-resistant Gram-negative and vancomycin-resistant Gram-positive strains were specifically detected.

**Conclusions:**

The LBJMR medium is a multipurpose selective medium which makes it possible to identify bacteria of interest from clinical samples and to isolate contaminated patients in hospital settings. This is a simple medium that could be easily used for screening in clinical microbiology laboratories.

**Electronic supplementary material:**

The online version of this article (10.1186/s12866-017-1128-x) contains supplementary material, which is available to authorized users.

## Background

The worldwide emergence of multidrug-resistant (MDR) bacteria represents a major public health issue. Controlling the spread of these bacteria relies upon both reducing the prescription of antibiotics and preventing transmission from carrier patients to others [1]. More specifically, this prevention targets the emerging carbapenemase-producing *Enterobacteriaceae* and vancomycin-resistant enterococci (VRE) strains, with the development of specific detection methods, mostly based on chromogenic and selective culture media [2].

The increase in infections due to carbapenemase-producing *Enterobacteriaceae* has led to the revival of colistin as a last-resort treatment [3]. Its widespread use, particularly in the livestock food in many countries [4], has inevitably led to the emergence of colistin-resistant strains over the past ten years [5, 6]. Until recently, all colistin resistance mechanisms which had been described were attributed to chromosomic mutations [7]. In China in 2015, Liu and colleagues were the first to report the plasmid-mediated colistin-resistance gene in animals and humans, which they named *mcr-1* [8]. This was followed by descriptions of the variants *mcr-1.2* and *mcr-1.3* [9, 10], *mcr-2*, *mcr-3*, *mcr-4* and *mcr-5* genes [11–14]. A significant number of human cases (in the majority of cases asymptomatic carriers) was described [15, 16]. Particularly, the worldwide analysis of colistin-resistant strains with an unknown mechanism allowed the detection of a consequent number of *mcr-1*-positive bacteria [17–19]. This mobile colistin resistance gene presents low resistance levels, with minimal inhibitory concentrations (MIC) of colistin around 4 μg/ml, which is close to the clinical breakpoint of colistin resistance (> 2 μg/mL), according to the European Committee on Antimicrobial Susceptibility Testing (EUCAST) [20]. Those data raised concerns about the detection and isolation of these pathogens, and the screening of the *mcr-1* gene into carbapenemase-producing bacteria has been added to recommendations for clinical microbiology laboratories in France [21]. While carbapenemase screening is well-defined with phenotypic and molecular techniques [22], there is a need for colistin-resistance screening tools [2]. Given the diversity of mechanisms of colistin resistance [23], phenotypic methods, such as chromogenic culture media [2, 24] are preferred for a rapid detection.

In addition to colistin-resistant *Enterobacteriaceae*, the detection of vancomycin-resistant enterococci isolates is of clinical concern. The prevalence of VRE strains is increasing in Europe, especially *Enterococcus faecium* [25] and has led to nosocomial infections in the United States [26], and their dissemination is associated with high mortality rates [27]. The development of an effective screening tool for the early detection of those multi-drug resistant pathogens has become a priority, in order to adapt treatment and isolate patients who are either infected or carriers.

For such purposes, we developed a polyvalent selective culture medium which can isolate both colistin-resistant Gram-negative bacteria, including *Enterobacteriaceae* strains harboring the *mcr-1* gene, and vancomycin-resistant Gram-positive bacteria. In this study, we evaluated the performances of this medium on a representative set of bacterial strains and fecal samples. This universal culture medium was named LBJMR, standing for Lucie Bardet-Jean-Marc Rolain, and has recently been patented [28].

## Methods

### Bacterial strains and samples cultured on LBJMR

A total of 143 bacterial strains were used in this study, of which 101 were *Enterobacteriaceae*: 75 with an acquired mechanism of resistance to colistin (including 32 strains harboring the *mcr-1* gene [6, 19, 29–37] and 43 to another resistance mechanism), six with an intrinsic colistin resistance mechanism [38], and 20 colistin-susceptible strains (Additional file 1: Table S1). This set also comprised 17 non-fermentative Gram-negative bacteria: ten were colistin-resistant strains [39] including one *Pseudomonas aeruginosa,* one *Acinetobacter baumannii* [39] and other pathogens that are frequently diagnosed in samples from patients with cystic fibrosis, and seven colistin-susceptible strains [40, 41]. Finally, 25 Gram-positive strains, including eight vancomycin-resistant enterococci [42], three intrinsically vancomycin-resistant genera and 14 vancomycin-susceptible strains [43] were also included in this study (Additional file 1: Table S1).

The colistin-resistance genes which were potentially involved were previously screened by RT-PCR for *Enterobacteriaceae* [6, 44], as were the vancomycin resistance genes *vanA* and *vanB* for the VRE strains [45]. MICs of colistin and vancomycin were determined using E-test® strips (BioMérieux, Marcy l’Étoile, France) for Gram-negative and Gram-positive strains, respectively, and MICs of daptomycin were determined for the four reference strains of *Enterococcus faecium* (DSM 17050, 25,698, 25,697 and 13,590), and were evaluated according to the EUCAST guidelines [20].

In addition, 68 samples were cultivated on LBJMR medium, including 66 stool samples (56 from humans and ten from chickens) that were previously screened for *mcr-1* by RT-PCR (56 positive and ten negative) and two clinical rectal swabs obtained from patients in the La Timone hospital, from which a vancomycin-resistant *E. faecium* VRE strain was isolated. Colonies with different morphologies isolated by culture of samples on LBJMR were isolated by subculture on Trypticase Soy Agar (TSA, Becton-Dickinson, Heidelberg, Germany), and the species were identified using Matrix-Assisted Laser Desorption-Ionization-Time Of Flight Mass Spectrometry (MALDI-TOF MS), as described previously [46].

### LBJMR development

In order to choose the most suitable medium for selecting colistin-resistant *Enterobacteriaceae*, including strains harboring the *mcr-1* gene, five commercial differential media usually used for the detection of *Enterobacteriaceae* were supplemented with a range of concentration of colistin sulfate salt (MP Biomedicals, Illkirch, France) from 0 to 32 μg/mL: Drigalski Lactose Agar (Biokar diagnostics, France), BBL™ Eosin Methylene Blue Agar (EMB; Becton-Dickinson, Heidelberg, Germany), Difco™ Violet Red Bile Agar (Becton-Dickinson, Heidelberg, Germany), BBL™ MacConkey Agar II (Becton-Dickinson, Heidelberg, Germany), and Difco™ Purple Agar Base (Becton-Dickinson, Heidelberg, Germany) supplemented with 7.5 g /L of glucose (MP Biomedicals, Illkirch, France). Four *Klebsiella pneumoniae,* including two colistin-resistant, and four *Escherichia coli,* including two which were colistin-resistant with the *mcr-1* gene, were inoculated at 10^6^ CFU on each prepared agar [19].

The Purple Agar Base supplemented with glucose (7.5 g/L), colistin sulfate (0, 4 and 8 μg/mL) and vancomycin (0 and 50 μg/mL) (Sandoz, Levallois-Perret, France) was evaluated with 24 representative strains, including 20 *Enterobacteriaceae* (five intrinsic genera, eight with acquired resistance and seven colistin-susceptible strains as control) and four Gram-positive strains susceptible to vancomycin, which were inoculated at 10^6^ CFU. This experiment was also conducted by replacing glucose with lactose (Laboratoires Humeau, La Chapelle-sur-Erdre, France). The optimum concentration of vancomycin for the medium composition was then determined using 20 Gram-positive strains representative of vancomycin resistance (three intrinsically-resistant genera and 17 enterococci, including seven VRE) and ten chicken stool samples from Algeria, five of which were positive for *mcr-1* by RT-PCR, and five of which were negative [19, 44].

### LBJMR evaluation

The sensitivity and specificity of the LBJMR medium to detect all colistin-resistant Gram-negative bacteria and all vancomycin-resistant Gram-positive bacteria were evaluated with all the strains listed in Additional file 1: Table S1 and samples listed in Additional file 1: Table S2, which were incubated on LBJMR in an aerobic atmosphere at 37 °C for between 24 and 48 h.

Inocula of the strains were prepared at 0.5 McFarland (corresponding to 1.5 × 10^8^ CFU/mL according to the EUCAST expert system) and serial 10-fold dilutions were then performed in Phosphate Buffer Saline. 10 μL of these suspensions were deposited on agars and simultaneously on TSA medium to determine the detection limits for each bacterium by assessing the viable concentration by colony count. Growth or inhibition of these microorganisms was observed after overnight incubation at 37 °C in an aerobic atmosphere. Those experiments were performed in duplicate.

Finally, as the LBJMR medium was developed so as to avoid *Proteus* swarming, *Proteus mirabilis* and *Proteus vulgaris* strains were inoculated alone or mixed with *E. coli* P10 (*mcr-1*) at the same concentration to evaluate the isolation ability of the LBJMR medium. Cultures were incubated at 37 °C for 72 h.

### Mcr-1 screening on stool samples

A total of 1052 stool samples were used in this study, 899 from humans from different regions of the world: Marseille (*n* = 212) and Angers (*n* = 128) in France, Thailand (*n* = 212), Laos (*n* = 189), Nigeria (*n* = 143), and Senegal (*n* = 15). 153 samples were from animals, including chickens from Algeria (*n* = 10), rodents from Cambodia (*n* = 21), and rodents (*n* = 60), pigs (*n* = 16) and goats (*n* = 46) from Laos.

The presence of the *mcr-1* gene in those stools was first screened using RT-PCR as previously described [44]. Briefly, the DNA of the stools was extracted, after pre-treatment by proteinase K and incubation at 95 °C, using Biorobot EZ1 Advanced XL (Qiagen, Hilden, Germany). Positive DNA was confirmed using standard PCR. Samples with positive DNA were then cultured on LBJMR, after a prior enrichment step performed by inoculating approximately 1 g of the sample in tryptic soy broth (TSB) medium (BioMérieux, Marcy l’Étoile, France) and incubating for 24 h at 37 °C. 100 μL of these liquid media were then plated on agars that were also incubated at 37 °C for 24 h.

For each Gram-negative isolate identified by MALDI-TOF MS, colistin MIC was determined with a colistin E-test® strip. For each colistin-resistant isolate, the presence of *mcr-1* was investigated by RT-PCR as previously described [44]. Susceptibility testing to 26 antibiotics was performed on *mcr-1-*positive strains using the disk diffusion method, and interpreted according to the EUCAST guidelines [20].

### Comparison of the LBJMR medium with other polymyxin-containing media

The sensitivity and specificity of the LBJMR medium was first compared to the BD™ Cepacia Medium (Cepacia, Becton-Dickinson, Heidelberg, Germany) medium with all the colistin-resistant *Enterobacteriaceae* strains listed in Additional file 1: Table S1. The LBJMR medium was then compared to the SuperPolymyxin medium with 103 bacterial strains, including 61 of the *Enterobacteriaceae*, 17 non-fermentative Gram-negative, and 25 Gram-positive strains (Additional file 1: Table S1). Other polymyxin-containing media were also compared concomitantly to the LBJMR medium, using EMB Agar (Sigma-Aldrich, Illkirch, Germany) as a control, with some of these strains as described below. Inocula were prepared following the same protocol as for the LBJMR evaluation.

Columbia Colistin Nalidixic Acid Agar +5% sheep blood (CNA) (BioMérieux, Marcy l’Étoile, France) and three mixed culture media: EMB with colistin sulfate (4 μg/mL) and vancomycin (50 μg/mL), LBJMR with Daptomycin (10 μg/ml, Novartis, Horsham, United Kingdom) in place of vancomycin and LBJMR with Amphotericin B (5 μg/ml, Bristol-Myers Squibb, Rueil-Malmaison, France) were concomitantly tested with 45 *Enterobacteriaceae* strains representative of colistin resistance (30 *mcr-1,* ten susceptible and five intrinsically resistant genera). LBJMR with Amphotericin B was also evaluated with the eight VRE strains. Finally, the LBJMR medium was compared to the SuperPolymyxin medium with 12 samples, including the two clinical rectal swabs from the La Timone hospital and ten human stools from Thailand, five of which had been identified as positive by culture for the detection of an isolate harboring the *mcr-1* gene, and five controls that were negative for *mcr-1* using RT-PCR.

### Statistical analysis

The data were analyzed using a Chi-test to compare the Cepacia and LBJMR media and a Student-t test for a pairwise comparison of SuperPolymyxin and LBJMR selective media for the detection of non-fermentative Gram-negative colistin-resistant strains. Significance was assessed at *p* < 0.05.

## Results

### LBJMR development

Because colistin is a cationic molecule, we looked for a medium which was deprived of any ions in its original composition and which was also deprived of any electrolytes to avoid *Proteus* spp. swarming. The optimal conditions for LBJMR medium (growth of colistin- and vancomycin-resistant strains and inhibition of colistin- or vancomycin susceptible strains) were obtained on Purple Agar Base with glucose (Additional file 1: Table S3), 4 μg/mL of colistin sulfate and 50 μg/ml of vancomycin (Additional file 1: Table S4). The culture of ten chicken stools from Algeria enabled the detection of *E. coli* strains harboring the *mcr-1* gene from three of the five samples which were positive for *mcr-1* by RT-PCR, including strain 235 which had previously been isolated on Cepacia medium [19] and no Gram-negative strains were detected from the five negative samples (Additional file 1: Table S2). A correlation was observed between the Ct-values that reflect the DNA concentration and the results of culture (Additional file 1: Table S2).

The final preparation of the LBJMR medium was as follows: 31 g/L of Purple Agar Base, 7.5 g/L of glucose, 4 μg/mL of colistin sulfate and 50 μg/mL of vancomycin. 7.5 g/L of lactose was used in place of glucose for the 24 strains initially tested and the same results were obtained. Glucose was selected because, combined with bromocresol purple, a pH indicator, it revealed the polyvalent capacity of the LBJMR medium with an easy and fast visualization of the species of interest in the clinical routine: both *Enterobacteriaceae* and enterococci gave yellow colonies, contrasting on the purple agar and exhibiting different sizes (2–3 mm and 0.1–1 mm, respectively).

### LBJMR evaluation

All the polymyxin-resistant *Enterobacteriaceae* strains (*n* = 81) tested were able to grow on the LBJMR medium, with the lowest detection limit (10^1^ CFU), including those with the *mcr-1* gene, as well as all the colistin-resistant non-fermentative Gram-negative (*n* = 10) and vancomycin-resistant Gram-positive strains (*n* = 11), with detection limits dependent on their MIC, as shown in Table 1. Meanwhile, all the bacteria susceptible to colistin or vancomycin were inhibited (not detected at 10^5^ CFU), giving rise to 100% sensitivity and specificity for the growth of Gram-negative colistin-resistant and Gram-positive vancomycin-resistant strains (Table 1). The swarming of *Proteus* sp. strains was fully inhibited on LBJMR, even after 48 h of incubation*.*

**Table 1:** Detection limits of targeted bacteria on LBJMR and SuperPolymyxin culture media

Bacterial strains			Detection limits on culture media (CFU):	
Gram-negatives		CT MIC	LBJMR	SuperPolymyxin
*Colistin-resistant Enterobacteriaceae*	***E. coli*** **SE65**	4	10^1^	10^1^
	***E. coli*** **117R**	4	10^1^	10^1^
	***E. coli*** **1R**	4	10^1^	10^1^
	***E. coli*** **1R 2104**	4	10^1^	10^1^
	***E. coli*** **44A**	4	10^1^	10^1^
	***E. coli*** **6R**	4	10^1^	10^1^
	***E. coli*** **85R**	4	10^1^	10^1^
	***E. coli*** **95R**	4	10^1^	10^1^
	***E. coli*** **96R**	4	10^1^	10^1^
	***E. coli*** **134R**	3	10^1^	10^1^
	***E. coli*** **143R**	3	10^1^	10^1^
	***E. coli*** **LH121**	16	10^1^	10^1^
	***E. coli*** **LH140** ^**1**^	12	10^1^	10^1^
	***E. coli*** **LH257**	12	10^1^	10^1^
	***E. coli*** **LH57** ^**1**^	8	10^1^	10^1^
	***E. coli*** **LH1**	6	10^1^	10^1^
	***E. coli*** **LH30**	6	10^1^	10^1^
	***E. coli*** **TH214**	6	10^1^	10^1^
	***E. coli*** **TH99**	4	10^1^	10^1^
	***E. coli*** **235**	4	10^1^	10^1^
	***E. coli*** **P6**	6	10^1^	10^1^
	***E. coli*** **P10**	4	10^1^	10^1^
	***E. coli*** **P17**	4	10^1^	10^1^
	*E. coli* FHA102^2^	12	10^1^	10^1^
	*E. coli* FHM19^3^	12	10^1^	10^1^
	*E. coli* FHA113^4^	12	10^1^	10^1^
	*E. coli* NH94^5^	12	10^1^	10^1^
	*E. coli* TH176	6	10^1^	10^1^
	***K. pneumoniae*** **FHA60**	8	10^1^	10^1^
	***K. pneumoniae*** **FHM128**	4	10^1^	10^1^
	***K. pneumoniae*** **119R**	3	10^1^	10^1^
	***K. pneumoniae*** **LH131** ^**6**^	32	10^1^	10^1^
	***K. pneumoniae*** **LH61** ^**7**^	24	10^1^	10^1^
	***K. pneumoniae*** **LH17**	12	10^1^	10^1^
	***K. pneumoniae*** **LH92**	12	10^1^	10^1^
	*K. pneumoniae* LB1^6^	32	10^1^	10^1^
	*P. mirabilis* FH112	>256	10^1^	10^1^
	*P. vulgaris* PV148	>256	10^1^	10^1^
	*P. alcalifaciens* TH44	>256	10^1^	10^1^
	*M. morganii* FM102	>256	10^1^	10^1^
	*S. marcescens* E13	128	10^1^	10^1^
*Others colistin-resistant*	*B. cepacia* FHM-BC1	64	10^3^	10^5^
	*B. cepacia* FHM-BC2	>256	10^1^	10^1^
	*A. xylosoxidans* FHM-AX	3	10^1^	>10^5^
	*S. maltophilia* FHM-SM	12	10^3^	>10^5^
	*I. limosus* FHM-IL	>256	10^4^	10^4^
	*P. pulmonicola* FHM-PP	>256	10^1^	10^3^
	*S. putrefaciens* FHM-SP	NC	10^3^	>10^5^
	*O. anthropi* FHM-OA	NC	10^2^	>10^5^
	*P. aeruginosa* FHM-PACOLR1	>256	10^1^	10^1^
	*A. baumannii* ABIsac_ColiR	8	10^2^	10^1^
*Colistin-susceptible*	*E. asburiae* P113	0,19	>10^5^	>10^5^
	*E. cloacae* NH151	0,50	>10^5^	>10^5^
	*E. cloacae* NH74	0,38	>10^5^	>10^5^
	*K. pneumoniae* CIP 82.91	0,125	>10^5^	>10^5^
	*K. pneumoniae* LB2	0,128	>10^5^	>10^5^
	*K. pneumoniae* TH20S	0,125	>10^5^	>10^5^
	*K. pneumoniae* TH28S	0,125	>10^5^	>10^5^
	*Proteus vulgaris* P100	0,94	>10^5^	>10^5^
	*S. enterica* 108R	1	>10^5^	>10^5^
	*S. enterica* 122R	0,5	>10^5^	>10^5^
	*E. coli* ATCC 25922 CIP 76.24	0,094	>10^5^	>10^5^
	*E. coli* 161	0,094	>10^5^	>10^5^
	*E. coli* 169	0,094	>10^5^	>10^5^
	*E. coli* FHM88S	0,125	>10^5^	>10^5^
	*E. coli* TH134S	0,094	>10^5^	>10^5^
	*E. coli* LH53S	0,094	>10^5^	>10^5^
	*E. coli* LH165S	0,074	>10^5^	>10^5^
	*E. coli* TH77S	0,064	>10^5^	>10^5^
	*E. coli* 282S	0,074	>10^5^	>10^5^
	*E. coli* FHM19S	0,125	>10^5^	>10^5^
	*P. aeruginosa* FHM-PA4	2	>10^5^	>10^5^
	*P. aeruginosa* FHM-PA5	0,50	>10^5^	>10^5^
	*P. aeruginosa* FHM-PA6	0,38	>10^5^	>10^5^
	*S. xianemensis* 111P	0,125	>10^5^	>10^5^
	*S. xianemensis* 111A	0,094	>10^5^	>10^5^
	*A. nosocomialis* ABG13S	0,064	>10^5^	>10^5^
	*A. pitti* MK	0,125	>10^5^	>10^5^
Gram-positives		VCN MIC		
*Vancomycin-resistant*	*E. faecium* DSM17050^a^	>256	10^1^	>10^5^
	*E. faecium* DSM13590^a^	>256	10^1^	>10^5^
	*E. faecium* DSM25698^a^	>256	10^2^	>10^5^
	*E. faecium* DSM25697^a^	>256	10^1^	>10^5^
	*E. faecium* FHMVRE1^a^	>256	10^1^	>10^5^
	*E. faecium* FHMVRE2^a^	>256	10^1^	>10^5^
	*E. faecium* VRE	24	10^3^	>10^5^
	*E. faecalis* JH2–2: Tn 1549^b^	64	10^2^	>10^5^
	*W. cibaria* P18A	>256	10^1^	>10^5^
	*W. cibaria* P18B	>256	10^1^	>10^5^
	*L. lactis* P18C	>256	10^1^	>10^5^
*Vancomycin-susceptible*	*S. aureus* CF_Marseille	1	>10^5^	>10^5^
	*E. faecium* TH26	0,75	>10^5^	>10^5^
	*E. faecium* 349	0,75	>10^5^	>10^5^
	*E. faecium* TH12	2	>10^5^	>10^5^
	*E. faecium* LH165	0,75	>10^5^	>10^5^
	*E. faecium* Al7	0,75	>10^5^	>10^5^
	*E. faecium* TH43	0,75	>10^5^	>10^5^
	*E. faecium* TH95	0,75	>10^5^	>10^5^
	*E. faecium* 282	0,75	>10^5^	>10^5^
	*E. faecalis* JH2–2: C2	1,5	>10^5^	>10^5^
	*E. faecalis* JH2–2S	1,5	>10^5^	>10^5^
	*E. gallinarum* SE3	1,5	>10^5^	>10^5^
	*E. casseliflavus* SE3	1,5	>10^5^	>10^5^
	*E. hirae* LH111	1	>10^5^	>10^5^

MIC of colistin (CT) and vancomycin (VCN) are given in μg/mL. *Enterobacteriaceae* with *mcr-1* gene are in bold font

Colistin-resistant strains with a mutation on another gene are indicated as follow: ^1^ *phoP* E375K, ^2^ *pmrB* A159V, ^3^ *pmrB* P7-Q12(del 6aa), ^4^ *pmrB* T156 K, ^5^ *pmrB* I92 insertion, ^6^ *mgrB* Stop, ^7^ *mgrB Sub,* ^8^ *pmrB T157P. Enterococci* strains with vancomycin-resistant genes are indicated as follows: ^a^ *vanA* and ^b^ *vanB*

Of the 1052 stools screened for *mcr-1* by RT-PCR, 66 were cultured on LBJMR, including 56 that were positive after 32 cycles of RT-PCR, as well as ten negative stool samples as controls. 16 cultures were positive on LBJMR medium, enabling the detection of 17 colistin-resistant *Enterobacteriaceae* strains harboring the *mcr-1* gene (colistin MICs ranging from 2 to 8 μg/mL): 15 were identified as *E. coli* and two as *K. pneumoniae* by MALDI-TOF MS (Additional file 1: Table S2)*.* Indeed, two bacterial species, *E. coli* and *K. pneumoniae*, were detected from the culture of the same sample (FHM128) (Additional file 1: Table S2). Five isolates were already known from a previous study on Cepacia medium [6, 19]. All the *E. coli* isolates from France were Extended-Spectrum Beta-Lactamase-producing strains (data not shown). In conclusion, the LBJMR medium enabled the isolation of new colistin-resistant strains harboring the *mcr-1* gene. The weak culture results compared to PCR could be explained by the length of storage of the tested samples (up to four years) at −80 °C, possibly inducing the death of bacteria while their DNA was still detectable [47]. However, it should be noted that, for some samples, we isolated another kind of strain, mostly Gram-positive bacteria which have an intrinsic resistance to vancomycin, largely *Pediococcus pentosaceus* and *Weissela cibaria*, as shown in Additional file 1: Table S2.

**Fig. 1:**
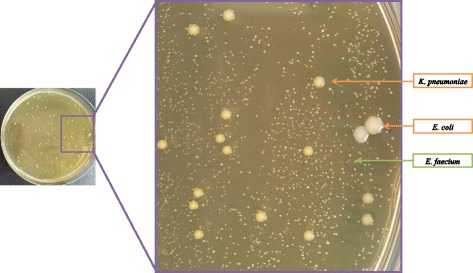
Aspect of the different types of colonies on LBJMR after culture of a clinical sample. *Enterobacteriaceae* exhibited yellow colonies that are larger (2–3 mm) than enterococci (0.1–1 mm)

Finally, the culture of two clinical samples from the hospital enabled the isolation of two vancomycin-resistant *E. faecium* isolates (VRE1 and VRE2). In addition, two colistin-resistant *Enterobacteriaceae* isolates were also detected in the VRE2 sample, identified as *K. pneumoniae* LB3 (MIC = 64 μg/mL) and *E. coli* LB4 (MIC = 16 μg/mL)*,* both negative for the *mcr-1* gene (Fig. 1).

### Comparison between LBJMR and other selective polymyxin-containing media

The LBJMR medium was more sensitive than the Cepacia and CNA media for detecting the strains exhibiting the *mcr-1* gene (Additional file 1: Table S5)*.* Indeed, only four of those strains could grow on the CNA medium, because it also contains nalidixic acid and fewer than half of them grew on the Cepacia medium. Comparison of the sensitivities of the LBJMR (100%) and Cepacia (47%) media using a Chi-square statistical test gave a significant *p*-value (<10^−4^).

LBJMR and SuperPolymyxin media presented the same sensitivity for *Enterobacteriaceae,* with the growth of all colistin-resistant strains (detection limit of 10^1^ CFU), including the 30 *mcr-*1 strains tested, and the inhibition of all susceptible strains (Table 1). In addition, the culture of ten human stools gave the same results: five were positive, with the detection of an *E. coli* positive for *mcr-1*, and the five controls remained negative (Additional file 1: Table S2). In contrast, the LBJMR medium showed a significantly higher sensitivity than the SuperPolymyxin medium for the detection of colistin-resistant non-fermentative Gram-negative strains, with a significant *p*-value of 0.019 (<0.01) with a pairwise comparison using a Student-t test (Table 1). More specifically, the LBJMR medium was able to detect those screened in the cystic fibrosis samples, such as *Burkholderia cepacia,* even at low concentrations, and could replace specific media such as the Cepacia medium for their isolation. The addition of amphotericin B or the replacement of vancomycin by daptomycin in the LBJMR medium did not affect the growth of *Enterobacteriaceae* strains (Additional file 1: Table S5). Finally, all the VRE strains were inhibited on the SuperPolymyxin medium because of the presence of daptomycin, while they were detected on the LBJMR medium (Table 1).

These results were confirmed by the culture of the two clinical samples from the hospital: vancomycin-resistant *E. faecium* could only grow on the LBJMR medium and not on the SuperPolymyxin medium for both samples, and the two *Enterobacteriaceae* from the VRE2 sample found on the LBJMR medium were also detected on the SuperPolymyxin medium.

Finally, the concomitant addition of colistin and vancomycin to EMB agar showed the systematic inhibition of *E. coli* strains harboring the *mcr-1* gene, corresponding to the tested strains with the lowest MIC.

## Discussion

In accordance with recommendations aiming to isolate patients carrying multi-drug resistant bacteria, it is becoming essential to detect colistin-resistant Gram-negative pathogens, particularly with the recent description of plasmid-mediated colistin-resistant genes among *Enterobacteriaceae* strains [8–12]. We do not currently know the potential consequences of patient colonization by those resistant bacteria, as it has already been demonstrated that acquisition of resistance is associated with a decrease in virulence [48–50], but it is necessary to implement efficient tools to prevent and monitor as closely as possible any epidemic outbreak and develop a rapid therapeutic strategy.

Currently, the only available method for detecting colistin resistance, according to joint EUCAST and Clinical Laboratory Standard Institute (CLSI) expert systems, is antibiotic susceptibility testing using broth microdilution, which is not suitable for daily screening in clinical microbiology laboratories. Current polymyxin-containing culture media are also not appropriate because of their high concentration of polymyxin. Indeed, they were not developed to isolate *Enterobacteriaceae* but to specifically detect bacteria that are intrinsically resistant to polymyxins, avoiding contaminants such as *Enterobacteriaceae* species. Thus, the establishment of an effective protocol to detect colistin resistance with the development of a reliable culture medium was necessary in clinical microbiology laboratory.

The significant number of strains tested on our selective medium showed that the LBJMR medium allows all colistin-resistant *Enterobacteriaceae* strains to grow, even those with a very low MIC for colistin and harboring the *mcr-1* gene, when all the susceptible strains were inhibited. Furthermore, the screening conducted on stool samples that were positive for *mcr-1* by PCR allowed the isolation of new *mcr-1* strains (Additional file 1: Table S2). The LBJMR medium could also detect colistin-resistant non-fermentative Gram-negative bacteria, including pathogens which are often found in patients suffering from cystic fibrosis (Table 1). We thereby created a universal culture medium, able to replace specific polymyxin-containing culture media usually used in routine diagnosis. All the vancomycin-resistant bacteria tested were detected at low concentrations on the LBJMR medium, including the VRE strains, when susceptible were inhibited. To attest this capacity, the LBJMR medium should be tested on more VRE strains and comparatively to VRE selective culture media.

The performances of the LBJMR medium are summarized in Table 2: samples can be cultured directly on the LBJMR selective medium, without a previous decontamination step, and colonies can be directly analyzed from the primary culture, without subcultures. Bacteria of interest are easily recognizable, as shown in Fig. 1: both colistin-resistant *Enterobacteriaceae* and vancomycin-resistant enterococci exhibit yellow colonies (2–3 mm and 0.1–1 mm respectively), on a purple agar base. Because LBJMR also permits the growth of intrinsic resistant bacteria, screening should be completed with a rapid identification using MALDI-TOF MS [51], directly from the LBJMR medium. Antibiotic susceptibility testing and PCR screening can be performed in the same time.

**Table 2:** Performance of LBJMR medium

Criteria	LBJMR medium
Isolates screened	Colistin-resistant Gram-negatives: - *Enterobacteriaceae*, including those harboring the *mcr-1* gene - Non-fermentative Gram-negative colistin-resistant strains, including those involves in cystic fibrosis samples Vancomycin-resistant Gram positives, including Enterococci
Aspect of colonies	Yellow on purple agar: 2–3 mm for *Enterobacteriaceae*, 0.1–1 mm for Enterococci
Incubation	Aerobic atmosphere, 37 °C, 24H (sterile at 48H)
Culture of samples	Direct on LBJMR, no previous decontamination
Isolates analysis	Colonies can be picked directly from primary cultures on LBJMR for analysis: - MALDI-TOF identification - Antibiotic Susceptibility testing - PCR screening for resistance genes.
Avoid contamination	- Inhibition of *Proteus* swarming - Inhibition of yeast possible by adding amphotericin B

Nordmann et al. developed a different selective medium called SuperPolymyxin, based on EMB agar and containing 3.5 μg/mL of colistin sulfate, 10 μg/mL of daptomycin and 5 μg/mL of amphotericin B [52], associated with a complementary phenotypic test, the rapid polymyxin NP [53], to screen for colistin-resistant Gram-negative bacteria [54]. These two media are differential and are able to recognize bacterial species based on color. Sensitivities for colistin-resistant *Enterobacteriaceae* were the same for the SuperPolymyxin and LBJMR media, but only the LBJMR medium could detect VRE strains (Table 1). Indeed, the SuperPolymyxin medium is composed of daptomycin and amphotericin B, which prevents the growth of contaminants including bacteria of interest such as VRE strains, because they are mostly susceptible to daptomycin. In our study, we tested stool samples and obtained some contaminants, including Gram-positive bacteria which were intrinsically resistant to vancomycin (Additional file 1: Table S2) or some yeasts.

More recently, the culture medium ChromAgar COL-APSE was developed and compared to the SuperPolymyxin medium [55]. The comparison of the LBJMR and the CHROMAgar COL-APSE performances have to be assessed. All those media should be also evaluated on heteroresistant strains, as they are difficult to detect and their frequence is widely underestimated [56].

The advantage of the LBJMR medium in terms of isolating vancomycin resistant enterococci can be also a disadvantage in a few cases, because Gram-positive bacteria which are intrinsically resistant to vancomycin bacteria, including *Pediococcus*, can also be isolated. In the LBJMR medium, Gram-positive bacteria appear with small colonies and a simple identification by MALDI-TOF can identify them. For the elimination of yeast in samples, amphotericin B can be added in our medium.

## Conclusion

The LBJMR medium is an adequate screening tool for all colistin-resistant isolates from clinical samples, independently of their resistance level or mechanism. LBJMR could be used in routine laboratory work to detect colistin-resistant and vancomycin-resistant bacteria, allowing for the direct analysis of colonies and, thus, the early isolation of contaminated patients in hospital settings. This medium is currently being investigated as a routine medium in our institute to determine its usefulness in detecting such bacteria.

## Additional file

Additional file 1: Table S1. List of the studied strains. The MIC of colistin (CT) or vancomycin (VCN) are indicated in μg/mL. **Table S2.** List of stools used and Gram-negative isolates detected on LBJMR. Positive isolates for *mcr-1* and their corresponding samples are in bold font. New isolates harboring *mcr-1* are underlined. Their MICs of colistin are indicated in μg/mL. (−) Negative (ND) fers to Not Done. **Table S3.** Comparison of different agar bases supplemented with different concentrations of colistin. Genes involved in colistin resistance are indicated in bold and colistin MICs are indicated in μg/mL. **Table S4.** Development of the selective medium for the detection of colistin-resistant strains. **Table S5.** Comparison of different polymyxin-containing media. Numbers indicate the total number of strains that grew on each culture media. (DOCX 99 kb)

## Abbreviations

CLSI: Clinical Laboratory Standard Institute
CNA: Colistin Nalidixic Acid
EMB: Eosin-Methylene Blue
EUCAST: European Committee on Antimicrobial Susceptibility Testing
MALDI-TOF MS: Matrix-Assisted Laser Desorption-Ionization-Time Of Flight Mass Spectrometry
MIC: Minimum Inhibitory Concentration
TSA: Trypticase Soya Agar
VRE: Vancomycin-Resistant Enterococci
