# Supplementary material for: LBJMR medium: a new polyvalent culture medium for isolating and selecting vancomycin and colistin-resistant bacteria
Source: BMC Microbiol. 2017 Nov 23;17:220. doi: 10.1186/s12866-017-1128-x (PMC5701432; doi:10.1186/s12866-017-1128-x)
Supplement: Additional file 1: Table S1. — List of the studied strains. The MIC of colistin (CT) or vancomycin (VCN) are indicated in μg/mL. Table S2. List of stools used and Gram-negative isolates detected on LBJMR. Positive isolates for mcr-1 and their corresponding samples are in bold font. New isolates harboring mcr-1 are underlined. Their MICs of colistin are indicated in μg/mL. (−) Negative (ND) fers to Not Done. Table S3. Comparison of different agar bases supplemented with different concentrations of colistin. Genes involved in colistin resistance are indicated in bold and colistin MICs are indicated in μg/mL. Table S4. Development of the selective medium for the detection of colistin-resistant strains. Table S5. Comparison of different polymyxin-containing media. Numbers indicate the total number of strains that grew on each culture media. (DOCX 99 kb) [file 12866_2017_1128_MOESM1_ESM.docx]

**Table S1. List of the studied strains.** The MIC of colistin (CT) or vancomycin (VCN) are indicated in µg/mL.

| **Gram-negative** | **Isolates** | **Origin** | **Samples** | **CT MIC** | **Resistance genes involved** | **References** |
| --- | --- | --- | --- | --- | --- | --- |
| ***Enterobacteriaceae* with an acquired resistance to colistin** | | | | | | |
| *Escherichia coli* | SE65 | Algeria | Human | 4 | *mcr-1* | [26] |
|  | 117R | Saudi arabia | Human | 4 | *mcr-1* | [29] |
|  | 1R | Saudi arabia | Human | 4 | *mcr-1* | [29] |
|  | 1R 2104 | Saudi arabia | Human | 4 | *mcr-1* | [29] |
|  | 44A | Saudi arabia | Human | 4 | *mcr-1* | [29] |
|  | 6R | Saudi arabia | Human | 4 | *mcr-1* | [29] |
|  | 85R | Saudi arabia | Human | 4 | *mcr-1* | [29,35] |
|  | 95R | Saudi arabia | Human | 4 | *mcr-1* | [29] |
|  | 96R | Saudi arabia | Human | 4 | *mcr-1* | [29] |
|  | 134R | Saudi arabia | Human | 3 | *mcr-1* | [29] |
|  | 143R | Saudi arabia | Human | 3 | *mcr-1* | [29] |
|  | LH121 | Laos | Human | 16 | *mcr-1* | [17] |
|  | LH140 | Laos | Human | 12 | *mcr-1* and *PhoQ* | [17] |
|  | LH257 | Laos | Human | 12 | *mcr-1* | [17] |
|  | LH57 | Laos | Human | 8 | *mcr-1* and *PhoQ* | [17] |
|  | LH1 | Laos | Human | 6 | *mcr-1* | [17] |
|  | LH30 | Laos | Human | 6 | *mcr-1* | [17] |
|  | TH214 | Thailand | Human | 6 | *mcr-1* | [17] |
|  | TH99 | Thailand | Human | 4 | *mcr-1* | [17] |
|  | FHA102 | France | Human | 12 | *pmrB* | [17] |
|  | FHM19 | France | Human | 12 | *pmrB* | [17] |
|  | FHA113 | France | Human | 12 | *pmrB* | [17] |
|  | NH94 | Nigeria | Human | 12 | *pmrB* | [17] |
|  | TH176 | Thailand | Human | 6 | Unknown | [17] |
|  | LB4 | France | Human | 16 | Unknown | This study |
|  | 235 | Algeria | Chicken | 4 | *mcr-1* | [17] |
|  | SA9 | Algeria | Chicken | 3 | *mcr-1* | This study |
|  | SE3 | Algeria | Chicken | 3 | *mcr-1* | This study |
|  | P6 | Laos | Pig | 6 | *mcr-1* | [17] |
|  | P10 | Laos | Pig | 4 | *mcr-1* | [17] |
|  | P17 | Laos | Pig | 4 | *mcr-1* | [17] |
| *Klebsiella pneumoniae* | FHA60 | France | Human | 8 | *mcr-1* | [6,28] |
|  | FHM128 | France | Human | 4 | *mcr-1* | [6,28] |
|  | 119R | Saudi arabia | Human | 3 | *mcr-1* | [29] |
|  | LH131 | Laos | Human | 32 | *mcr-1* and *mgrB* | [6,28] |
|  | LH61 | Laos | Human | 24 | *mcr-1* and *mgrB* | [6,28] |
|  | LH17 | Laos | Human | 12 | *mcr-1* and *pmrB* | [6,28] |
|  | LH92 | Laos | Human | 12 | *mcr-1* | [6,28] |
|  | LB1 | France | Human | 32 | *mgrB* | [33] |
|  | FHM169 | France | Human | 8 | *mgrB* | [6] |
|  | LH12 | Laos | Human | 32 | *mgrB* | [6] |
|  | LH74 | Laos | Human | 12 | *mgrB* | [6] |
|  | NH53 | Nigeria | Human | 4 | *mgrB* | [6] |
|  | TH20 | Thailand | Human | 32 | *mgrB* | [6] |
|  | TH196 | Thailand | Human | 8 | *mgrB* | [6] |
|  | TH28 | Thailand | Human | 8 | *mgrB* | [6] |
|  | TH213 | Thailand | Human | 12 | *mgrB* | [6] |
|  | LH70 | Laos | Human | 12 | *pmrA* | [6] |
|  | TH54 | Thailand | Human | 16 | *pmrB* | [6] |
|  | TH224 | Thailand | Human | 4 | *pmrB* | [6] |
|  | TH205 | Thailand | Human | 64 | *PhoQ* | [6] |
|  | FHM120 | France | Human | 64 | Unknown | [6] |
|  | FHA105 | France | Human | 24 | Unknown | [6] |
|  | FHM77 | France | Human | 8 | Unknown | [6] |
|  | LH140 | Laos | Human | 16 | Unknown | [6] |
|  | LH94 | Laos | Human | 16 | Unknown | [6] |
|  | LH375 | Laos | Human | 12 | Unknown | [6] |
|  | LH102 | Laos | Human | 8 | Unknown | [6] |
|  | TH34 | Thailand | Human | 24 | Unknown | [6] |
|  | TH68 | Thailand | Human | 64 | Unknown | [6] |
|  | TH176 | Thailand | Human | 12 | Unknown | [6] |
|  | TH21 | Thailand | Human | 12 | Unknown | [6] |
|  | TH114 | Thailand | Human | 8 | Unknown | [6] |
|  | TH164 | Thailand | Human | 6 | Unknown | [6] |
|  | TH166 | Thailand | Human | 3 | Unknown | [6] |
|  | LB3 | France | Human | 64 | Unknown | This study |
| *Klebsiella oxytoca* | FHA41 | France | Human | 12 | *mgrB* | [6,32] |
|  | FHA124 | France | Human | 6 | Unknown | [6] |
| *Enterobacter aerogenes* | EA1509E | France | Human | >256 | *pmrA* | [31] |
| *Enterobacter asburiae* | LH74 | Laos | Human | > 256^#^ | Unknown | [34] |
| *Enterobacter cloacae* | NH52 | Nigeria | Human | > 256^#^ | Unknown | [34] |
|  | NH131 | Nigeria | Human | 64^#^ | Unknown |  |
|  | NH132 | Nigeria | Human | > 256^#^ | Unknown |  |
| *Salmonella enterica* | 65RC | Saudi arabia | Human | 16 | Unknown | [30] |
|  | 100RC3 | Saudi arabia | Human | 8 | Unknown | [30] |
| ***Enterobacteriaceae* with an intrinsic resistance to colistin** | | | | | | |
| *Proteus mirabilis* | FH112 | France | Human | >256 | Intrinsic |  |
| *Proteus vulgaris* | PV148 | France | Human | >256 | Intrinsic |  |
| *Providencia alcalifaciens* | TH44 | Thailand | Human | >256 | Intrinsic |  |
| *Providencia rettgeri* | H1734 | France | Human | >256 | Intrinsic | [36] |
| *Morganella morganii* | FM102 | France | Human | >256 | Intrinsic |  |
| *Serratia marcescens* | E13 | Laos | Environnement | 128 | Intrinsic |  |
| **Non –fermentative resistant to colistin** | | | | | | |
| *Burkholderia cepacia* | FHM-BC1 | France | Human | 24 | Unknown |  |
|  | FHM-BC2 | France | Human | >256 | Unknown |  |
| *Achromobacter xylosoxidans* | FHM-AX | France | Human | 3 | Unknown |  |
| *Stenotrophomonas maltophilia* | FHM-SM | France | Human | 12 | Unknown |  |
| *Inquilinus limosus* | FHM-IL | France | Human | >256 | Intrinsic |  |
| *Pandoraea pulmonicola* | FHM-PP | France | Human | >256 | Unknown |  |
| *Shewanella putrefaciens* | FHM-SP | France | Human | NC | Intrinsic |  |
| *Ochromobacter anthropi* | FHM-OA | France | Human | NC | Intrinsic |  |
| *Pseudomonas aeruginosa* | FHM-PACOLR1 | France | Human | >256 | Unknown |  |
| *Acinetobacter baumannii* | ABIsac_ColiR | France | Human | 8 | Unknown | [37] |
| **Susceptible to colistin** | | | | | | |
| *Enterobacter asburiae* | P113 | France | Human | 0,19 | Susceptible |  |
| *Enterobacter cloacae* | NH141 | Nigeria | Human | 0,50 | Susceptible | [34] |
|  | NH74 | Nigeria | Human | 0,38 | Susceptible |  |
| *Klebsiella pneumoniae* | ATCC 13883CIP 82.91 |  | Human | 0,125 | Susceptible |  |
|  | LB2 | France | Human | 0,128 | Susceptible | [33] |
|  | TH20S | Thailand | Human | 0,125 | Susceptible |  |
|  | TH28S | Thailand | Human | 0,125 | Susceptible |  |
| *Proteus vulgaris* | P100 | Algeria | Human | 0,94 | Susceptible |  |
| *Salmonella enterica* | 108R | Saudi arabia | Human | 1 | Susceptible | [30] |
|  | 122R | Saudi arabia | Human | 0,5 | Susceptible | [30] |
| *Escherichia coli* | ATCC 25922 CIP 76.24 |  |  | 0,094 | Susceptible |  |
|  | 161 | Algeria | Chicken | 0,094 | Susceptible |  |
|  | 169 | Algeria | Chicken | 0,094 | Susceptible |  |
|  | FHM88S | France | Human | 0,125 | Susceptible |  |
|  | TH134S | France | Human | 0,094 | Susceptible |  |
|  | LH53S | Laos | Human | 0,094 | Susceptible |  |
|  | LH165S | Laos | Human | 0,074 | Susceptible |  |
|  | TH77S | Thailand | Thailand | 0,064 | Susceptible |  |
|  | 282S | Algeria | Chicken | 0,074 | Susceptible |  |
|  | FHM19S | France | Human | 0,125 | Susceptible |  |
| *Pseudomonas aeruginosa* | FHM-PA4 | France | Human | 2 | Susceptible |  |
|  | FHM-PA5 | France | Human | 0,50 | Susceptible |  |
|  | FHM-PA6 | France | Human | 0,38 | Susceptible |  |
| *Shewanella xianemensis* | 111P | France | Human | 0,125 | Susceptible |  |
|  | 111A | France | Human | 0,094 | Susceptible |  |
| *Acinetobacter nosocomialis* | ABG13S | France | Human | 0,064 | Susceptible | [38] |
| *Acinetobacter pitti* | MK | France | Human | 0,125 | Susceptible | [39] |

| **Gram-positive** | **Isolates** | **Origin** | **Samples** | **VCN**  **MIC** | **Resistance mechanism** | **Ref** |
| --- | --- | --- | --- | --- | --- | --- |
| **Vancomycin-resistant *Enterococci* (VRE)** | | | | | | |
| *Enterococcus faecium* | DSM17050 | Germany | Human | >256 | *vanA* |  |
|  | DSM13590 | Germany | Human | >256 | *vanA* |  |
|  | DSM25698 | South Korea | Human | >256 | *vanA* |  |
|  | DSM25697 | South Korea | Human | >256 | *vanA* |  |
|  | FHMVRE1 | France | Human | >256 | Susceptible | This study |
|  | FHMVRE2 | France | Human | >256 | Unknown | This study |
|  | VRE | France | Human | 24 | Unknown |  |
| *Enterococcus faecalis* | JH2-2:Tn 1549 | France | Human | 64 | *vanB* | [40] |
| **Intrinsic resistance to vancomycin** | | | | | | |
| *Weissela cibaria* | P18A | Laos | Pig | >256 | Unknown |  |
| *Weissela confusa* | P18B | Laos | Pig | >256 | Unknown |  |
| *Leuconostoc lactis* | P18C | Laos | Pig | >256 | Unknown |  |
| **Susceptible to vancomycin** | | | | | | |
| *Staphylococcus aureus* | CF_Marseille | France | Human | 1 | Susceptible | [41] |
| *Enterococcus faecium* | TH26 | Thailand | Human | 0,75 | Susceptible |  |
|  | 349 | Algeria | Chicken | 0,75 | Susceptible |  |
|  | TH12 | Thailand | Human | 2 | Susceptible |  |
|  | LH165 | Laos | Human | 0,75 | Susceptible |  |
|  | Al7 | Algeria | Chicken | 0,75 | Susceptible |  |
|  | TH43 | Thailand | Human | 0,75 | Susceptible |  |
|  | TH95 | Thailand | Human | 0,75 | Susceptible |  |
|  | 282 | Algeria | Chicken | 0,75 | Susceptible |  |
| *Enterococcus faecalis* | JH2-2 : C2 | Thailand | Human | 1,5 | Susceptible | [40] |
|  | JH2-2S | Thailand | Human | 1,5 | Susceptible | [40] |
| *Enterococcus gallinarum* | SE3 | Algeria | Chicken | 1,5 | Susceptible |  |
| *Enterococcus casseliflavus* | SE3 | Algeria | Chicken | 1,5 | Susceptible |  |
| *Enterococcus hirae* | LH111 | Laos | Human | 1 | Susceptible |  |

^#^Heteroresistance

**Table S2. List of samples cultured on LBJMR medium and identified isolates**. Positive isolates for *mcr-1* and their corresponding samples are in bold, and the newly isolated are underlined. MICs of colistin (Col MIC) are in µg/mL. MICs of vancomycin are indicated in brackets and are in µg/mL.

| **Samples and results of qPCR**  **for *mcr-1* gene** (cycles) | | | **Gram-negative isolates**  **and colistin MIC** (µg/mL) | | **Gram-positives and yeast isolates** |
| --- | --- | --- | --- | --- | --- |
|  | PE1 | PE2 | Species | MIC |  |
| **Human samples from France** | | |  | |  |
| **FHA60** | 27.38 | 28.63 | ***K. pneumoniae***  *M. morganii* | 8  >256 | *Lactobacillus sakei*  *Candida albicans* |
| **FHM128** | 18.1 | 15.9 | ***K. pneumoniae*** | 4 | - |
|  |  |  | ***E. coli*** | 6 |  |
| **FHM157** | 17.17 | 15.44 | *E. coli* | 16 | - |
|  |  |  | ***E. coli*** | 6 |  |
| **FHM66** | 19.08 | 16.91 | ***E. coli* 1** | 6 | - |
|  |  |  | ***E. coli* 2** | 6 |  |
| VRE1 | ND | ND | **-** | | *Enterococcus faecium* (>256)  *Candida albicans* |
| VRE2 | ND | ND | *E. coli* | 16 | *Enterococcus faecium* (>256) |
|  |  |  | *K. pneumoniae* | 64 |  |
| **Human samples from Laos** | | |  | |  |
| **LH213** | 31.14 | 28.71 | ***E. coli***  *A. guillouae* | 2  2 | *Pediococcus pentosaceus*  *Weissella cibaria* |
| **LH259** | 27.22 | 22.58 | ***E. coli* 1** | 3 | *Weissella cibaria* |
|  |  |  | ***E. coli* 2** | 3 | - |
|  |  |  | ***E. coli* 3** | 3 |  |
| **LH345** | 31.68 | 31.02 | *E. coli* 1 | 3 | - |
|  |  |  | ***E. coli* 2** | 3 |  |
|  |  |  | ***E. coli* 4** | 3 |  |
| LH102^*^ | 32.21 | 32.06 | - | | - |
| LH106 | 26.25 | 24.18 | - | | - |
| LH111 | 19.41 | 17.73 | - | | *Pediococcus pentosaceus*  *Enterococcu hirae* |
| LH113 | 31.28 | 29.7 | - | | - |
| LH114 | 28.41 | 27.34 | - | | *Pediococcus pentosaceus* |
| LH121^*^ | 34.19 | 28.97 | - | | *Pediococcus pentosaceus* |
| LH136 | 31.81 | 27.78 | - | | - |
| LH15 | 31.46 | 31.52 | - | | *Enterococcus faecium*  *Pediococcus pentosaceus* |
| LH165 | 27.88 | 26.74 | - | | *Enterococcus faecium*  (2) |
| LH172 | 33.74 | 28.37 | - | | - |
| LH183 | 31.05 | 29.26 | - | | - |
| LH248 | 27.79 | 26.38 | - | | - |
| LH252 | 31.07 | 30.13 | - | | *Weissella cibaria* |
| LH30^*^ | 31.44 | 30.85 | - | | *Weissella cibaria*  *Weissella confusa* |
| LH312 | 26.51 | 25.03 | - | | - |
| LH328 | 29.37 | 27.97 | - | | *Weissella cibaria* |
| LH355 | 28.26 | 27.8 | - | | *Bacillus cereus*  *Enterococcus faecium* |
| LH48 | 31.05 | 30.99 | - | | - |
| LH53 | 32.09 | 30.67 | - | | *Weissella falsenii* [Pseudochrobactrum](https://en.wikipedia.org/wiki/Pseudochrobactrum_saccharolyticum) saccharolyticum *Comamonas kerstersii*  *Enterococcus hirae* |
| LH57^*^ | 31.06 | 31.11 | - | | - |
| LH90 | 29.59 | 28.53 | - | | - |
| LH93 | 29.22 | 29.3 | - | | - |
| LH94^*^ | 28.88 | 28.4 | - | | *Weissella cibaria*  *Lactococcus garviae*  *Pediococcus pentosaceus* |
| LH95 | 28.66 | 27.48 | - | | - |
| LH98 | 26.11 | 25.88 | - | | - |
| **Human samples from Thailand** | | |  | |  |
| **TH134** | 29.91 | 28.95 | ***E. coli*** | 4 | *Weissella cibaria* |
| **TH169** | 29.86 | 28.83 | ***E. coli* 1** | 3 | - |
|  |  |  | ***E. coli* 2** | 3 |  |
|  |  |  | *E. coli* 5 | 2 |  |
| **TH44** | 24.8 | 23.16 | ***E. coli* 1** | 6 | - |
|  |  |  | ***E. coli* 2** | 8 |  |
| **TH99** | 31.07 | 29.6 | ***E. coli* 6** | 4 | *Enterococcus hirae* (2) |
| **TH66** | 29.67 | 28.71 | *E. coli* 1 | 4 | - |
|  |  |  | ***E. coli* 2** | 3 |  |
|  |  |  | *P. rettgeri* | >256 |  |
|  |  |  | *P.alcalifaciens* | >256 |  |
|  |  |  | *E. cloacae* | 64^#^ |  |
|  |  |  | *E. asburiae*  *P. vulgaris* | 128^#^  >256 |  |
| TH33 | 32.57 | 30.47 | *E. coli* 1 | 2 | *Pediococcus pentosaceus* |
|  |  |  | *E. coli* 2 | 3 | *Weissella cibaria* |
| TH118 | 28.4 | 27.26 | - | | *Weissella cibaria*  *Pediococcus pentosaceus*  *Enterococcus faecium* |
| TH12 | 30.54 | 29.58 | - | | *Enterococcus faecium* |
| TH123 | 33.85 | 31.65 | - | | *Pediococcus pentosaceus*  *Weissella cibaria* |
| TH136 | 25 | 23.58 | - | | *Pediococcus pentosaceus*  *Weissella cibaria* |
| TH16 | 33.26 | 31.28 | - | | - |
| TH174 | 26.25 | 25.09 | - | | - |
| TH206 | 32.92 | 31.56 | - | | *Pediococcus pentosaceus*  *Enterococcus hirae* |
| TH214^*^ | 27.44 | 26.25 | - | | - |
| TH78 | 31.01 | 29.55 | - | | *Weissella cibaria* |
| TH82 | 24.43 | 22.63 | - | | *Weissella cibaria*  *Weissella paramesenteroides* |
| TH92 | 31.96 | 29.76 | - | | - |
| TH77 | - | | - | | - |
| TH26 | - | | - | | *Pediococcus pentosaceus* |
| TH1 | - | | - | | *Lactobacillus paracasei*  *Lactobacillus plantarum* |
| TH19 | - | | - | | *Pediococcus pentosaceus* |
| TH43 | - | | - | | *Pediococcus pentosaceus* |
| **Pig samples from Laos** | | |  | |  |
| P10 | 32.72 | 32.01 | *E. coli* | 6 | *Weissella falsenii*  *Leuconostoc mesenteroides* |
| **P6** | 33.04 | 32.25 | ***E. coli***  *S. marcescens*  *C. gleum* | 2  >256  >256 | *Weissella cibaria, Sphingobacterium multivorum,* |
| **Chicken samples from Algeria** | | |  | |  |
| **235** | 26.32 | 26.56 | ***E. Coli*** | 4 | - |
| **SE3** | 30.56 | 31 | ***E. Coli*** | 3 | - |
| **SA9** | 29.04 | 29.8 | ***E. Coli*** | 4 | *Pediococcus acidilactici Enterococcus faecium* (0,5) |
| Al7 | 34 | 34.78 | *-* | | *Enterococcus avium* (0,75) *Enterococcus faecium* (0,75) |
| SE1 | 32.89 | 33.04 | *-* | | *Enterococcus casseliflavus* |
| 270 | - | | *-* | | *Lactobacillus salivarius Enterococcus faecium Pediococcus acidilactici* |
| 282 | - | | *-* | | *Enterococcus gallinarum Enterococcus avium* |
| 307 | - | | *-* | | *Enterococcus faecalis* |
| 229 | - | | *-* | | - |
| 349 | - | | *-* | | *Proteus mirabilis* yeast |

^#^heteroresistant ^*^a colistin-resistant *Enterobacteriaceae* was isolated in a previous study on Cepacia medium

**Table S3. Comparison of different agar bases supplemented with different concentrations of colistin.** Genes involved in colistin resistance are indicated in bold and colistin MICs are indicated in µg/mL.

| **Strains and colistin susceptibility** | | | **Agar bases and concentration of colistin (µg/mL)** | | | | | | | | | | | | | | | | | | | | | | | | | | | | | |
| --- | --- | --- | --- | --- | --- | --- | --- | --- | --- | --- | --- | --- | --- | --- | --- | --- | --- | --- | --- | --- | --- | --- | --- | --- | --- | --- | --- | --- | --- | --- | --- | --- |
| Isolates | | MIC | Purple Agar Base* | | | | | | MacConkey | | | | | | Drigalski | | | | | | EMB | | | | | | VRBL | | | | | |
|  |  |  | 0 | 2 | 4 | 8 | 16 | 32 | 0 | 2 | 4 | 8 | 16 | 32 | 0 | 2 | 4 | 8 | 16 | 32 | 0 | 2 | 4 | 8 | 16 | 32 | 0 | 2 | 4 | 8 | 16 | 32 |
| ***E. coli*** | P17 (***mcr-1*)** | 4 | + | + | + | + | + | - | + | + | + | + | + | + | + | + | + | + | - | - | + | + | + | + | - | - | + | + | + | - | - | - |
|  | TH214 (***mcr-1*)** | 6 | + | + | + | + | + | + | + | + | + | + | + | + | + | + | + | + | + | + | + | + | + | - | - | - | + | + | + | + | - | - |
|  | 161 | 0,128 | + | - | - | - | - | - | + | + | + | - | - | - | + | - | - | - | - | - | + | - | - | - | - | - | + | - | - | - | - | - |
|  | 169 | 0,094 | + | - | - | - | - | - | + | + | - | - | - | - | + | - | - | - | - | - | + | - | - | - | - | - | + | - | - | - | - | - |
| ***K. pneumoniae*** | TH28 (***mgrB***) | 8 | + | + | + | + | + | + | + | + | + | + | + | + | + | + | - | - | - | - | + | + | + | + | + | - | + | - | - | - | - | - |
|  | LB1 (***mgrB***) | 32 | + | + | + | + | + | + | + | + | + | + | + | + | + | + | + | + | - | - | + | + | + | + | + | + | + | + | + | - | - | - |
|  | TH20S | 0,128 | + | - | - | - | - | - | + | + | + | + | - | - | + | - | - | - | - | - | + | - | - | - | - | - | + | - | - | - | - | - |
|  | LB2 | 0,128 | + | - | - | - | - | - | + | + | - | - | - | - | + | - | - | - | - | - | + | - | - | - | - | - | + | - | - | - | - | - |

^*^Glucose was added as fermentative substract

(+) Bacterial growth (-) No growth

**Table S4. Development of the selective medium for the detection of colistin-resistant strains.**

| **Bacterial strains** | **Colistin MIC** (µg/mL) | **Purple Agar Base + glucose supplemented with : colistin + vancomycin (µg/mL)** | | | | | |
| --- | --- | --- | --- | --- | --- | --- | --- |
|  |  | **0 + 0** | **0 + 50** | **4 + 0** | **4 + 50** | **8 + 0** | **8 + 50** |
| **Intrinsic resistance to colistin** | | | | | | | |
| *M. morganii* PM102 | >256 | + | + | + | + | + | + |
| *P. mirabilis* FH112 | >256 | + | + | + | + | + | + |
| *P. vulgaris* PV148 | >256 | + | + | + | + | + | + |
| *P. alcalifaciens* TH44 | >256 | + | + | + | + | + | + |
| *S. marcescens* E13 | 128 | + | + | + | + | + | + |
| **Acquired resistance to colistin** | | | | | | | |
| *E. aerogenes* 1509 | >256 | + | + | + | + | + | + |
| *E. asburiae* LH74 | >256 | + | + | + | + | + | + |
| *E. cloacae* NH52 | >256 | + | + | + | + | + | + |
| *K. pneumoniae* LH140 | 96 | + | + | + | + | + | + |
| *K. pneumoniae* TH224 | 4 | + | + | + | + | + | + |
| *E. coli* LH257 | 6 | + | + | + | + | + | + |
| *E. coli* P17 | 4 | + | + | + | + | + | + |
| *S. enterica* 100RC3 | 8 | + | + | + | + | + | + |
| **Susceptible to colistin** | | | | | | | |
| *K. pneumonia* TH20 | 0,125 | + | + | - | - | - | - |
| *P. vulgaris* P100 | 0,94 | + | + | - | - | - | - |
| *S. enterica* 122R | 0,5 | + | + | - | - | - | - |
| *E. asburiae* P113 | 0,19 | + | + | - | - | - | - |
| *E. cloacae* NH151 | 0,50 | + | + | - | - | - | - |
| *E. cloacae* NH74 | 0,38 | + | + | - | - | - | - |
| *E. coli* 169 | 0,096 | + | + | - | - | - | - |
| **Gram-positive** |  |  |  |  |  |  |  |
| *Staphylococcus aureus* | >256 | + | - | + | - | + | - |
| *Enterococcus faecium* | >256 | + | - | + | - | + | - |
| *Enterococcus faecalis* | >256 | + | - | + | - | + | - |
| *Enterococcus gallinarum* | >256 | + | - | + | - | + | - |

(+) Growth (-) No growth

**Table S5. Comparison of different polymyxin-containing media.** Numbers indicate the total of strains that have grown on each culture media.

| **Bacterial strains** | **Total** | **LBJMR** | **SP^a^** | **Cepacia** | **CNA^b^** | **(1)** | **(2)** | **(3)** | **EMB^c^** |
| --- | --- | --- | --- | --- | --- | --- | --- | --- | --- |
| ***Acquired resistance to colistin with* mcr-1 *gene*** | | | | | | | | | |
| *E. coli* | 23 | 23 | 23 | 7 | 4 | 23 | 23 | 0 | 23 |
| *K. pneumoniae* | 7 | 7 | 7 | 7 | 0 | 7 | 7 | 7 | 7 |
| **Intrinsic resistance to colistin** | | | | | | | | | |
| *M. morganii* | 1 | 1 | 0 | 1 | 0 | 1 | 1 | 1 | 1 |
| *S. marcescens* | 1 | 1 | 1 | 1 | 0 | 1 | 1 | 1 | 1 |
| *P. mirabilis* | 1 | 1 | 1 | 1 | 0 | 1 | 1 | 1 | 1 |
| *P. vulgaris* | 1 | 1 | 1 | 1 | 0 | 1 | 1 | 1 | 1 |
| *P. alcalifaciens* | 1 | 1 | 0 | 0 | 0 | 1 | 1 | 1 | 1 |
| **Colistin-susceptible** | | | | | | | | | |
| *E. coli* | 2 | 0 | 0 | 0 | 0 | 0 | 0 | 0 | 0 |
| *K. pneumoniae* | 3 | 0 | 0 | 0 | 0 | 0 | 0 | 0 | 0 |
| *S. enterica* | 2 | 0 | 0 | 0 | 0 | 0 | 0 | 0 | 0 |
| *E. cloacae* | 1 | 0 | 0 | 0 | 0 | 0 | 0 | 0 | 0 |
| *E. asburiae* | 1 | 0 | 0 | 0 | 0 | 0 | 0 | 0 | 0 |
| *P. vulgaris* | 1 | 0 | 0 | 0 | 0 | 0 | 0 | 0 | 0 |

**^a^SP : SuperPolymyxin**

**^b^CNA : Colistin - Nalidixic Acid**

**^c^EMB : Eosin - Methylene Blue Agar**

**(1) Purple Agar Base + glucose + colistin + daptomycin**

**(2) Purple Agar Base + glucose + colistin + vancomycin + amphotericin B**

**(3) EMB + colistin + vancomycin**

**Table S6. Comparison of the antimicrobial composition of the different polymyxin-containing media.** (B) Polymyxin B, (C) Colistin, (AB) Amphotericin B, (A) Anisomycin, (CH) Cycloheximid, (MB) Methylen Blue, (N) Nystatin.

| Targeted bacteria | Culture media | Antimicrobial agents (µg/mL) against : | | | | References |
| --- | --- | --- | --- | --- | --- | --- |
|  |  | **Gram-negative** | | **Gram-positive** | **Yeast** |  |
|  |  | **Polymyxins** | **Others** |  |  |  |
| Colistin-resistant and Vancomycin-resistant | LBJMR^a^ | 4 (C) |  | Vancomycin 50 |  | This study |
| Colistin-resistant *Enterobacteriaceae* | SuperPolymyxin | 3,5 (C) |  | Daptomycine 10  MB 65  Eosin 400 | 5 (AB) | Nordmann *et al.,* 2016 |
| *Neisseria sp.* | Martin-Lewis Agar | 7,5 (C) | Trimethoprim 5 | Vancomycin 4 | 20 (A) | Martin and Lewis, 1977 |
|  | Thayer-Martin Agar | 7,5 (C) |  | Vancomycin 3 | 2,57 (N) | Thayer and Martin, 1966 |
|  | MTM^b^ Agar | 7,5 (C) | Trimethoprim 5 | Vancomycin 3 | 2,57 (N) | Martin and Lester, 1971 |
|  | NYC^c^ Agar | 7,5 (C) | Trimethoprim 3 | Vancomycin 2 | 20 (A) | Fauer *et al.*, 1973 |
| *Bulkhoderia cepacia* | Cepacia Medium | 35,7 (B) |  | Ticarcilline 100 |  | Gilligan *et al.,* 1985 |
|  | OFPBL^d^ Agar | 35,7 (B) |  | Bacitracine 2,7 |  | *Welch,* 1987 |
|  | Bulkhoderia Cepacia Agar | 17.8 (B) | Gentamicin 5 | Ticarcilline 100 |  | Gillian *et al.,* 1985 |
|  | Bulkhoderia Cepacia Selective Agar | 71.4 (B) | Gentamicin 10 | Vancomycin 2.5 |  | Henry, 1997 |
| *Streptococcus sp.*  and Gram-positives | CNA^e^ | 10 (C) | Nalidixic acid 10 |  |  | Ellner *et al.,* 1966 |
| *Legionella sp.* | BCYE^f^ Selective Agar with: | |  |  |  |  |
|  | - GPVC^g^ | 9,4 (B) | Glycin 3000 | Vancomycin 1 | 80 (CH) | Dennis *et al.*, 1984 |
|  | - CCVC^h^ | 16 (C) |  | Vancomycin 0,5  Cefalotin 4 | 80 (CH) | Bopp *et al*., 1981 |
|  | - GPVA^i^ | 11,9 (B) | Glycin 3000 | Vancomycin 1 | 80 (A) | Ta *et al.*, 1995 |
|  | - PAV^j^ | 4.76 (B) |  | Vancomycin 0,5 | 80 (A) | Stout *et al.,* 1982 |
|  | - PAC^k^ | 9.52 (B) |  | Cefamandol 2 | 80 (A) | Edelstein, 2007 |
|  | - DGVP^l^ | 9.4 (B) | Glycin 3000 | Vancomycin 1 |  | Murray, 2007 |
| *Campylobacter sp.* | Campylobacter Agar : |  |  |  |  |  |
|  | - Butzler | 0.3 (C) | Novobiocin 5 | Cephazolin 15  Bacitracin 337.8 | 50 (CH) | Butzler, 1973 |
|  | - Skirrow | 0.3 (B) | Trimethoprim 5 | Vancomycin 10 |  | Skirrow, 1977 |
|  | - Blaser-Wang | 0.3 (B) | Trimethoprim 5 | Vancomycin 10  Cefalotin 15 | 2 (AB) | Blaser *et al*., 1978 |
|  | - Preston | 0.3 (B) | Trimethoprim 5 | Rifampicin 5 | 50 (CH) | Bolton and Robertson, 1982 |
| *Brucella spp.* | Brucella selective medium | 1 (B) |  | Bacitracin 500 | 100 (CH) | Jones and Brinley, 1958 |
| *Vibrio sp.* | CPC^m^ | 66.34 (C)  11.9 (B) |  |  |  | Vanderzant and Splittstoesser, 1992 |
| *Listeria monocytogenes* | Oxford medium | 20 (C) | Fosfomycin 10 | Cefotetan 2  Acriflavin 5 | 400 (CH) | Curtis *et al.*, 1989 |
|  | Modified Oxford medium | 10 (C) |  | Moxalactam 20 |  | Lee *et al.,* 1989 |
| *Listeria spp.* | PALCAM^n^ | 10 (B) |  | Ceftazidim 8  Acriflavin 5 |  | van Nerren *et al.,* 1989 |
| *Bacillus cereus* | MYP^o^ | 11.9 (B) |  |  |  | Mossel, 1967 |
| *Mycobacteriaceae* | Middlebrook 7H11 Agar | 2.38 (B) | Trimethoprim 20 | Carbenicillin 50 | 20 (AB) | Cohn *et al,* 1968 |
| *Clostridium perfringens* | SPS^p^ Agar | 10 (B) | Sulfadiazin 120 |  |  | Angelotti *et al,* 1962 |
|  | TSN^q^ Agar | 20 (B) | Néomycin 50 |  |  | Marshall *et al.*, 1965 |
|  | SFP^r^ Agar | 3.57 (B) | Kanamycin 12 |  |  | Shahidi and Ferguson, 1971 |

^a^LBJMR : Our culture medium, ^b^MTM : Modified Thayer-Martin, NYC^c^: New York City, ^d^OFPBL : Oxidation/Fermentation, Polymyxin B, Bacitracin, Lactose, ^e^CNA : Colistin, Nalidixic acid, ^f^BCYE : Buffered Charcoal, Yeast Extract, ^g^GPVC : Glycin, Polymyxin B, Vancomycin et Cycloheximid, ^h^CCVC : Cephalotin, Colistin, Vancomycin et Cycloheximid, ^i^GPVA : Glycin, Polymyxin B, Vancomycin et Anisomycin, ^j^PAV : Polymyxin B, Anisomycin, Vancomycin, ^k^PAC : Polymyxin B, Anisomycin, Céfamandol, ^l^DGVP : Dyes, Glycin, Vancomycin, Polymyxin B, ^m^CPC : Cellobiose, Polymyxin B, Colistin, ^n^PALCAM : Polymyxin B, Acriflavin, Lithium, Ceftazidim, Esculine, Mannitol, ^o^MYP : Mannitol, Egg Yolk, Polymyxin B, ^p^SPS : Sulfite, Polymyxin B, Sulfadizin, ^q^TSN : Trypticase , Sulfite, Néomycin, ^r^SFP : Shahidi Ferguson Perfringens.
